# Supplementary material for: Improving type 2 diabetes polygenic risk scores by incorporating rare, low-frequency, and population-specific variants
Source: medRxiv. 2025 Nov 28:2025.11.24.25340878. Preprint. [Version 1] doi: 10.1101/2025.11.24.25340878 (PMC12676392; doi:10.1101/2025.11.24.25340878)
Supplement: Supplement 1 [file media-1.docx]

**Supplementary Material**

| **Supplementary Figures** | **1** |
| --- | --- |
| **Cohort acknowledgements and funding** | **5** |
| **Genes and Health Research Team Authorship for Scientific Publications** | **7** |

**Supplementary Figures**

**Supplementary Fig. 1 |** Mock table of effect sizes for each LD clumping parameter. When a variant is determined to be in LD with another variant for a given LD clumping parameter, it is given a weight of 0 and therefore would not contribute to the calculation of PRS for the given variant weight-set specific PRS.

**Supplementary Fig 2 | PRS distribution for carriers of individual rare variants and predictive performance for samples enriched with carriers of rare variants in AFR.** A) Variants and associated MAFs, ORs, SEs, p-values, found by meta-analysis. Number of carriers in validation cohort. B) Ridgeline plot showing PRS distributions for carriers of rare variants for CTSLEB and PRS-CS comparisons created with the same meta-analysis. Variant, number of carriers in validation cohort, and OR being a variant carrier, as determined by the meta-analysis, are written to the left of each distribution set. Non-carrier distributions are at the bottom of the plot. C) Mean incremental AUC (iAUC) with 95% confidence interval for samples enriched with carriers. For variants with < 30 carriers in validation cohort, sample size is 4 x number of carriers. Otherwise, sample size is 2x number of carriers. Non-carriers are randomly selected and predictive performance for each sample set is tested over 100 iterations for each variant. Colors represent PRS used: purple for CTSLEB, green for PRS-CS (HM3), bright blue for PRS-CS (TAGIT), muted blue for D-PRISM PRS.

**Supplementary Fig 3 | PRS distribution for carriers of individual rare variants and predictive performance for samples enriched with carriers of rare variants in AMR.** A) Variants and associated MAFs, ORs, SEs, p-values, found by meta-analysis. Number of carriers in validation cohort. B) Ridgeline plot showing PRS distributions for carriers of rare variants for CTSLEB and PRS-CS comparisons created with the same meta-analysis. Variant, number of carriers in validation cohort, and OR being a variant carrier, as determined by the meta-analysis, are written to the left of each distribution set. Non-carrier distributions are at the bottom of the plot. C) Mean incremental AUC (iAUC) with 95% confidence interval for samples enriched with carriers. For variants with < 30 carriers in validation cohort, sample size is 4 x number of carriers. Otherwise, sample size is 2x number of carriers. Non-carriers are randomly selected and predictive performance for each sample set is tested over 100 iterations for each variant. Colors represent PRS used: purple for CTSLEB, green for PRS-CS (HM3), bright blue for PRS-CS (TAGIT), muted blue for D-PRISM PRS.

**Supplementary Fig. 4 | PRS distribution for carriers of individual rare variants and predictive performance for samples enriched with carriers of rare variants in EUR.** A) Variants and associated MAFs, ORs, SEs, p-values, found by meta-analysis. Number of carriers in validation cohort. B) Ridgeline plot showing PRS distributions for carriers of rare variants for CTSLEB and PRS-CS comparisons created with the same meta-analysis. Variant, number of carriers in validation cohort, and OR being a variant carrier, as determined by the meta-analysis, are written to the left of each distribution set. Non-carrier distributions are at the bottom of the plot. C) Mean incremental AUC (iAUC) with 95% confidence interval for samples enriched with carriers. For variants with < 30 carriers in validation cohort, sample size is 4 x number of carriers. Otherwise, sample size is 2x number of carriers. Non-carriers are randomly selected and predictive performance for each sample set is tested over 100 iterations for each variant. Colors represent PRS used: purple for CTSLEB, green for PRS-CS (HM3), bright blue for PRS-CS (TAGIT), muted blue for D-PRISM PRS.

**Cohort acknowledgements and Funding**

All Of Us Research Program (AOU) is supported by the National Institutes of Health, Office of the Director: Regional Medical Centers: 1 OT2 OD026549; 1 OT2 OD026554; 1 OT2 OD026557; 1 OT2 OD026556; 1 OT2 OD026550; 1 OT2 OD 026552; 1 OT2 OD026553; 1 OT2 OD026548; 1 OT2 OD026551; 1 OT2 OD026555; IAA #: AOD 16037; Federally Qualified Health Centers: HHSN 263201600085U; Data and Research Center: 5 U2C OD023196; Biobank:1 U24 OD023121; The Participant Center: U24 OD023176; Participant Technology Systems Center: 1 U24 OD023163; Communications and Engagement: 3 OT2 OD023205; 3 OT2 OD023206; and Community Partners: 1 OT2 OD025277; 3 OT2 OD025315; 1 OT2 OD025337; 1 OT2 OD025276. In addition, the All of Us Research Program would not be possible without the partnership of its participants.

Estonian Biobank (ESTBB) was funded by the Estonian Research Council Grant IUT20-60, IUT24-6, PRG687, and the European Union through the European Regional Development Fund Project No. 2014-2020.4.01.15-0012 GENTRANSMED.

FinnGen study (FINNGEN) is a large-scale genomics initiative that has analyzed over 500,000 Finnish biobank samples and correlated genetic variation with health data to understand disease mechanisms and predispositions. The project is a collaboration between research organisations and biobanks within Finland and international industry partners. We want to acknowledge the participants and investigators of the FinnGen study.

Resource for Genetic Epidemiology on Adult Health and Aging (GERA) was supported by a grant (RC2 AG033067; PIs Schaefer and Risch) awarded to the Kaiser Permanente Research Program on Genes, Environment, and Health (RPGEH) and the UCSF Institute for Human Genetics. The RPGEH was supported by grants from the Robert Wood Johnson Foundation, the Wayne and Gladys Valley Foundation, the Ellison Medical Foundation, Kaiser Permanente Northern California, and the Kaiser Permanente National and Northern California Community Benefit Programs.

Genes and Health. Genes & Health is/has recently been core-funded by Wellcome (WT102627, WT210561), the Medical Research Council (UK) (M009017, MR/X009777/1, MR/X009920/1), Higher Education Funding Council for England Catalyst, Barts Charity (845/1796), Health Data Research UK (for London substantive site), and research delivery support from the NHS National Institute for Health Research Clinical Research Network (North Thames). Genes & Health is/has recently been funded by Alnylam Pharmaceuticals, Genomics PLC; and a Life Sciences Industry Consortium of AstraZeneca PLC, Bristol-Myers Squibb Company, GlaxoSmithKline Research and Development Limited, Maze Therapeutics Inc, Merck Sharp & Dohme LLC, Novo Nordisk A/S, Pfizer Inc, Takeda Development Centre Americas Inc. We thank Social Action for Health, Centre of The Cell, members of our Community Advisory Group, and staff who have recruited and collected data from volunteers. We thank the NIHR National Biosample Centre (UK Biocentre), the Social Genetic & Developmental Psychiatry Centre (King’s College London), Wellcome Sanger Institute, and Broad Institute for sample processing, genotyping, sequencing and variant annotation. This work uses data provided by patients and collected by the NHS as part of their care and support. This research utilised Queen Mary University of London’s Apocrita HPC facility, supported by QMUL Research-IT, <http://doi.org/10.5281/zenodo.438045>. We thank: Barts Health NHS Trust, NHS Clinical Commissioning Groups (City and Hackney, Waltham Forest, Tower Hamlets, Newham, Redbridge, Havering, Barking and Dagenham), East London NHS Foundation Trust, Bradford Teaching Hospitals NHS Foundation Trust, Public Health England (especially David Wyllie), Discovery Data Service/Endeavour Health Charitable Trust (especially David Stables), Voror Health Technologies Ltd (especially Sophie Don), NHS England (for what was NHS Digital) - for GDPR-compliant data sharing backed by individual written informed consent. Most of all we thank all of the volunteers participating in Genes & Health.

Mexican Biobank (MXBB) was supported by Mexico’s CONACYT (Grant number FONCICYT/50/2016; PI Moreno-Estrada), and the Newton Fund through the UK Medical Research Council (Grant number MR/N028937/1; PI Moreno-Estrada) to genetically characterize the population-based cohort derived from the National Health Survey 2000 (ENSA2000). The resulting ENSA Genomics Consortium acknowledges the seminal effort of Dr. Jaime Sepúlveda, the Mexican Ministry of Health, and the National Institute of Public Health, in the design and implementation of the ENSA2000 survey from which genomic data were generated for the MXB Project. Members of the ENSA Genomics Consortium are also acknowledged for biobank maintenance, sample selection and processing of materials contributed to the MXBB.

Mass General Brigham Biobank (MGBB) acknowledges the Partners HealthCare System for support of the MGB biobank and MGB patients for providing samples, genomic data, and health information data, as well as research support by NIDDK K24 DK110550 (to J.C.F.), K24 DK080140 (to J.B.M.) and NIDDK K23DK114551 (to M.S.U).

MyCode (Geisinger) recruitment and exome sequencing were funded through a partnership between Geisinger and the Regeneron Genetics Center. We thank the participants and providers for their contributions to this study.

Slim Initiative for Genomic Medicine in the Americas (SIGMA). This work was conducted as part of the Slim Initiative for Genomic Medicine, a joint U.S.-Mexico project funded by the Carlos Slim Health Institute. The UNAM/INCMNSZ diabetes study was supported by Consejo Nacional de Ciencia y Tecnología grants 138826, 128877, CONACyT- SALUD 2009-01-115250, and a grant from Dirección General de Asuntos del Personal Académico, UNAM, IT 214711. The Diabetes in Mexico Study was supported by Consejo Nacional de Ciencia y Tecnología grant 86867 and by Instituto Carlos Slim de la Salud, A.C. The Mexico City Diabetes Study was supported by National Institutes of Health (NIH) grant R01HL24799 and by the Consejo Nacional de Ciencia y Tenologia grants: 2092, M9303, F677-M9407, 251M, and 2005-C01-14502, SALUD 2010-2-151165. The Multiethnic Cohort was supported by NIH grants CA164973, CA054281, and CA063464.

UK Biobank (UKBB) analyses were conducted using the UK Biobank resource under applications 236, 9161, and 10035. This research was supported by the British Heart Foundation (grant SP/13/2/30111). Large-scale comprehensive genotyping of UK Biobank for cardiometabolic traits and diseases: UK CardioMetabolic Consortium (UKCMC).

Wellcome Trust Case Control Consortium (WTCCC) analysis and genotyping was supported by: Wellcome Trust funding 090367, 098381, 090532, 083948, 085475, 101630, and 203141; MRC (G0601261); EU (Framework 7) HEALTH-F4-2007-201413; and NIDDK DK098032 and U01-DK105535.

**Genes & Health Research Team authorship for Scientific Publications**

Eamonn Maher e.maher@aston.ac.uk Aston University

Shabana Chaudhary s.chaudhary@qmul.ac.uk Blizard Institute, Queen Mary University of London

Joseph Gafton j.gafton@qmul.ac.uk Blizard Institute, Queen Mary University of London

Karen A Hunt k.a.hunt@qmul.ac.uk Blizard Institute, Queen Mary University of London

Shapna Hussain shapna.hussain@qmul.ac.uk Blizard Institute, Queen Mary University of London

Kamrul Islam k.islam@qmul.ac.uk Blizard Institute, Queen Mary University of London

Mohammed Bodrul Mazid m.b.mazid@qmul.ac.uk Blizard Institute, Queen Mary University of London

Elizabeth Owor e.owor@qmul.ac.uk Blizard Institute, Queen Mary University of London

Jessry Russell jessry.russell@qmul.ac.uk Blizard Institute, Queen Mary University of London

Nishat Safa n.safa@qmul.ac.uk Blizard Institute, Queen Mary University of London

John Solly j.solly@qmul.ac.uk Blizard Institute, Queen Mary University of London

Marie Spreckley m.spreckley@qmul.ac.uk Blizard Institute, Queen Mary University of London

David A Van Heel d.vanheel@qmul.ac.uk Blizard Institute, Queen Mary University of London

Jan Whalley j.whalley@qmul.ac.uk Blizard Institute, Queen Mary University of London

Ishevanhu Zengeya i.zengeya@qmul.ac.uk Blizard Institute, Queen Mary University of London

Emily Mantle e.mantle@qmul.ac.uk Blizard Institute, Queen Mary University of London

Shaheen Akhtar shaheen.akhtar@bthft.nhs.uk Bradford Teaching Hospitals NHS Foundation Trust

Samina Ashraf samina.ashraf@bthft.nhs.uk Bradford Teaching Hospitals NHS Foundation Trust

Dan Mason dan.mason@bthft.nhs.uk Bradford Teaching Hospitals NHS Foundation Trust

John Wright john.wright@bthft.nhs.uk Bradford Teaching Hospitals NHS Foundation Trust

Daniel MacArthur d.macarthur@garvan.au.org Garvan Institute

Michael Simpson michael.simpson@kcl.ac.uk King's College London

Richard C Trembath richard.trembath@kcl.ac.uk King's College London

Gerome Breen gerome.breen@kcl.ac.uk Kings College London

Raymond Chung raymond.chung@kcl.ac.uk Kings College London

Sang Hyuck Lee sang_hyuck.lee@kcl.ac.uk Kings College London

Omar Asgar omar.asghar1@nhs.net Manchester University Hospitals

Joanne Harvey joanne.henry@nihr.ac.uk Manchester University Hospitals

Karen Tricker karen.tricker@mft.nhs.uk Manchester University Hospitals

Caroline Winckley caroline.winckley@nihr.ac.uk Manchester University Hospitals

Hanifa Khatun hanifa.khatun@mft.nhs.uk Manchester University Hospitals

Amna Asif amna.asif@mft.nhs.uk Manchester University Hospitals

Claudia Langenberg claudia.langenberg@qmul.ac.uk Precision Healthcare University Research Institute, Queen Mary University of London

Grainne Colligan grainnec@safh.org.uk Social Action for Health (charity)

Ceri Durham cerid@safh.org.uk Social Action for Health (charity)

| Bill Newman | william.newman@manchester.ac.uk University of Manchester |
| --- | --- |
| Ahsan Khan | cllrahsan.khan@walthamforest.gov.uk Waltham Forest Council |
| Hilary Martin | hilary.martin@qmul.ac.uk Wellcome Sanger Institute |
| Teng Heng | th13@sanger.ac.uk Wellcome Sanger Institute |
| Matt Hurles | meh@sanger.ac.uk Wellcome Sanger Institute |
| Vivek Iyer | vvi@sanger.ac.uk Wellcome Sanger Institute |

Georgios Kalantzis gk18@sanger.ac.uk Wellcome Sanger Institute

Vladimir Ovchinnikov vo3@sanger.ac.uk Wellcome Sanger Institute

| Iaroslav Popov ip13@sanger.ac.uk Wellcome Sanger Institute  Klaudia Walter kw8@sanger.ac.uk Wellcome Sanger Institute  Panos Deloukas p.deloukas@qmul.ac.uk William Harvey Research Institute, Queen Mary University of London | |
| --- | --- |
| David Collier | d.j.collier@qmul.ac.uk William Harvey Research Institute, Queen Mary Universityof London |
| Ana Angel of London | a.cristinaangelgarcia@qmul.ac.uk Wolfson Institute of Population Health, Queen Mary University |
| Saeed Bidi London | saeed.bidi@qmul.ac.uk Wolfson Institute of Population Health, Queen Mary University of |
| Fabiola Eto | f.eto@qmul.ac.uk Wolfson Institute of Population Health, Queen Mary University of London |
| Sarah Finer London | s.finer@qmul.ac.uk Wolfson Institute of Population Health, Queen Mary University of |
| Chris Griffiths  London | c.j.griffiths@qmul.ac.uk Wolfson Institute of Population Health, Queen Mary University of |
| Sam Hodgson | s.hodgson@qmul.ac.uk Wolfson Institute of Population Health, Queen Mary University of |

London

Benjamin M Jacobs b.jacobs@qmul.ac.uk Wolfson Institute of Population Health, Queen Mary University of London

Rohini Mathur r.mathur@qmul.ac.uk Wolfson Institute of Population Health, Queen Mary University of

London

Caroline Morton c.morton@qmul.ac.uk Wolfson Institute of Population Health, Queen Mary University of London

Asma Qureshi asmaa.qureshi@qmul.ac.uk Wolfson Institute of Population Health, Queen Mary University of London

Stuart Rison s.rison@qmul.ac.uk Wolfson Institute of Population Health, Queen Mary University of

London

Annum Salman a.salman@qmul.ac.uk Wolfson Institute of Population Health, Queen Mary University of

London

Miriam Samuel m.samuel@qmul.ac.uk Wolfson Institute of Population Health, Queen Mary University of London

Moneeza K Siddiqui moneeza.siddiqui@qmul.ac.uk Wolfson Institute of Population Health, Queen Mary University of London

| Daniel Stow London | d.stow@qmul.ac.uk Wolfson Institute of Population Health, Queen Mary University of |
| --- | --- |
| Sabina Yasmin of London | sabina.yasmin@qmul.ac.uk Wolfson Institute of Population Health, Queen Mary University |
| Julia Zöllner  London | j.zollner@qmul.ac.uk Wolfson Institute of Population Health, Queen Mary University of |
| Sheik Dowlut London | s.dowlut@qmul.ac.uk Wolfson Institute of Population Health, Queen Mary University of |
